# UNTOLD STORIES OF AGING – FOSTERING INTERGENERATIONAL DIALOGUE THROUGH AN ART-BASED COMMUNITY ACTION PROJECT

**DOI:** 10.1093/geroni/igae098.3920

**Published:** 2024-12-31

**Authors:** Sarah Jen

**Affiliations:** University of Kansas, Lawrence, Kansas, United States

## Abstract

Individuals who can imagine their aging futures are better prepared for the aging process. However, stories of later life often go untold, limiting public understanding of this complex phase of life. The Untold Stories of Aging Project was created to combat societal ageism through encouraging the creation of aging-focused art and intergenerational dialogue. Between 2021 - 2024, we have amassed a digital art exhibition including over 50 pieces of artwork from over 40 artists. Seven individual artists or groups have received awards for their submissions, honoring the evocative impact of their pieces, their connections to aging-related insights, and their creativity and originality. We present findings from interviews with awardees who discussed their artistic process, lessons learned about their own aging futures, and the intergenerational dialogue surrounding their pieces. Artists often cited intergenerational dialogue or the importance of family relationships as the motivation for their pieces. For example, one 26-year-old chose to challenge ageist stereotypes through her art following a conversation with her mother who described being perceived as “incapable” as she aged. Older artists shared how meaningful it was to be recognized as artists, with one 90-year-old stating, “It’s thrilling to know that something we are doing in such a common and ordinary way is impressive to anybody.” Through this project, artists have cultivated space for older adults to share their stories and legacies, illustrating how art and surrounding dialogue can combat societal ageism, foster intergenerational healing, and inspire audiences to consider and plan for their own aging futures.

